# Mammalian Bcnt/Cfdp1, a potential epigenetic factor characterized by an acidic stretch in the disordered N-terminal and Ser^250^ phosphorylation in the conserved C-terminal regions

**DOI:** 10.1042/BSR20150111

**Published:** 2015-07-14

**Authors:** Shintaro Iwashita, Takehiro Suzuki, Takeshi Yasuda, Kentaro Nakashima, Taiichi Sakamoto, Toshiyuki Kohno, Ichiro Takahashi, Takayasu Kobayashi, Yoshiko Ohno-Iwashita, Shinobu Imajoh-Ohmi, Si-Young Song, Naoshi Dohmae

**Affiliations:** * Faculty of Pharmacy, Iwaki Meisei University, Iwaki 970-8551, Japan; † Center for Sustainable Resource Science, RIKEN, Wako 351-0198, Japan; ‡ Research Center for Radiation Emergency Medicine, National Institute of Radiological Sciences, Chiba 263-8555, Japan; § Institute of Neuroscience, Tokushima Bunri University, Sanuki 769-2193, Japan; ║ Mitsubishi Kagaku Institute of Life Sciences, Machida 194-0031, Japan; ¶ Tsukuba Primate Research Center, National Institute of Biomedical Innovation, Tsukuba 305-0843, Japan; * *Department of Project Program, Institute of Development, Aging and Cancer, Tohoku University, Sendai 980-8575, Japan; † †Laboratory Center for Proteomics Research, Institute of Medical Science, The University of Tokyo, Tokyo 108-8639, Japan

**Keywords:** Bucentaur (BCNT) superfamily, disordered protein, mass spectroscopy, post-translational modification (PTM), sodium dodecyl sulfate/polyacrylamide gel electrophoresis (SDS/PAGE) mobility, the conserved C-terminal region of Bcnt/Cfdp1 (BCNT-C) domain

## Abstract

The BCNT (Bucentaur) superfamily is classified by an uncharacteristic conserved sequence of ∼80 amino acids (aa) at the C-terminus, BCNT-C (the conserved C-terminal region of Bcnt/Cfdp1). Whereas the yeast Swc5 and *Drosophila* Yeti homologues play crucial roles in chromatin remodelling organization, mammalian Bcnt/Cfdp1 (craniofacial developmental protein 1) remains poorly understood. The protein, which lacks cysteine, is largely disordered and comprises an acidic N-terminal region, a lysine/glutamic acid/proline-rich 40 aa sequence and BCNT-C. It shows complex mobility on SDS/PAGE at ∼50 kDa, whereas its calculated molecular mass is ∼33 kDa. To characterize this mobility discrepancy and the effects of post-translational modifications (PTMs), we expressed various deleted His–Bcnt in *E. coli* and HEK cells and found that an acidic stretch in the N-terminal region is a main cause of the gel shift. Exogenous BCNT/CFDP1 constitutively expressed in HEK clones appears as a doublet at 49 and 47 kDa, slower than the protein expressed in *Escherichia coli* but faster than the endogenous protein on SDS/PAGE. Among seven *in vivo* phosphorylation sites, Ser^250^, which resides in a region between disordered and ordered regions in BCNT-C, is heavily phosphorylated and detected predominantly in the 49 kDa band. Together with experiments involving treatment with phosphatases and Ser^250^ substitutions, the results indicate that the complex behaviour of Bcnt/Cfdp1 on SDS/PAGE is caused mainly by an acidic stretch in the N-terminal region and Ser^250^ phosphorylation in BCNT-C. Furthermore, Bcnt/Cfdp1 is acetylated *in vitro* by CREB-binding protein (CBP) and four lysine residues including Lys^268^ in BCNT-C are also acetylated *in vivo*, revealing a protein regulated at multiple levels.

## Abstract (precis)

We characterized the mammalian Bcnt/Cfdp1 (Bucentaur/craniofacial developmental protein 1) protein, a potential epigenetic factor, by showing that an acidic stretch in the N-terminal region and Ser^250^ phosphorylation in the C-terminal region are critical for its anomalous SDS/PAGE mobility.

## INTRODUCTION

Chromatin dynamics are pivotal aspects of gene regulation mechanisms in eukaryotes. A main issue is how the canonical histone H2A-H2B dimer is converted into various histone H2A variant-H2B dimers accompanied by common and/or unique chromatin-related components [1,2]. These changes vary depending on the developmental stage, the tissues involved and the responsiveness to both intra- and extracellular stresses. In order to characterize more precisely the dynamics of the chromatin complex, which provides platform(s) determining the timing and degree of gene expression, further information about the known and still unknown factors that interact with the complex, as well as innovative experimental approaches are required. One potential ‘orphan’ factor is the BCNT (Bucentaur) superfamily of proteins, a long-neglected class of essential proteins [3]. Mammalian Bcnt/Cfdp1 (craniofacial developmental protein 1; hereafter Bcnt) was first recognized as an orthologue of a bovine brain protein with a molecular mass of 97 kDa (p97Bcnt), a paralogue in Ruminantia and distributes widely in animals and plants [3–6].

This superfamily is classified by a conserved sequence of ∼80 amino acids (aa) at the C-terminus called BCNT-C (the conserved C-terminal region of Bcnt/Cfdp1). The yeast homologue, Swc5, is a unique component of the chromatin remodelling SWR1 complex that functions in the ATP-dependent histone exchange process [7]. The *Drosophila* homologue, Yeti, is essential for development by providing chaperon-like activity in higher chromatin organization [8]. Furthermore, the chicken homologue, CENP-29, has been identified as a centromere protein [9]. These results indicate that the BCNT superfamily has evolved as a chromatin-related functional molecule. On the other hand, mammalian Bcnt is poorly characterized, but localizes mainly in the cytosol as a tight protein complex [6], although a nuclear localization [3] and association with chromatin-related proteins [10] have also been reported. This discrepancy suggests the possibility that changes in the protein resulting from post-translational modifications (PTMs) may determine its cellular localization [11]. Thus a precise characterization of the protein is strongly required in order to clarify its functional role.

The protein comprises an acidic N-terminal region of ∼175 aa, a lysine/glutamic acid/proline-rich 40 aa region [called IR (intramolecular repeat)] and BCNT-C and is a typical intrinsically disordered protein [3,6] that appears heterogeneous on SDS/PAGE at ∼50 kDa despite its calculated molecular mass of ∼33 kDa. Since PTMs might be involved in its formation of complexes with various cellular component(s) for the proper function of this kind of disordered protein [12,13], we explored the molecular determinant(s) that cause the complex behaviour on SDS/PAGE including the effects of PTMs.

## EXPERIMENTAL

### Chemicals

Trichostatin A and okadaic acid were obtained from Wako Pure Chemical Industries. The protease inhibitor cocktail was from Nacalai Tesque and the protein phosphatase inhibitor cocktail was from Sigma. Nicotinamide was from Kanto Chemical.

### Plasmid construction and isolation

Human *BCNT* cDNA (IMAGE 3449836) was obtained from Open BioSystems and bovine *Bcnt* cDNA was prepared as described previously [14]. Two variant bovine *Bcnt* cDNAs, one that codes the 175 aa N-terminal region (corresponding to GenBank Accession number BAC11952) and another lacking the IR region (GenBank:AB846663), were identified during the preparation of bovine full-length cDNA. Target DNA fragments with both 5′- and 3′-restriction sites, for plasmid construction, were amplified by PCR using DNA polymerase (KOD-Plus-version 2, Toyobo). The PCR products were cloned once into a TA-vector (TArget Clone-Plus, Toyobo) for DNA sequencing and their fragments were inserted into pCold II (Takara Bio Inc.) through the NdeI/XhoI sites or into pCMV6-AN-His (OriGene Technologies) through the SgfI/MluI sites. Human BCNT deleted, swapped and Ser^250^ site-directed mutants were constructed according to the manufacturer's instructions (QuickChange Multi site-Directed Mutagenesis kit, Agilent Technologies). A plasmid encoding the human N-terminal region of 104 aa plus valine was prepared by PstI digestion of human *BCNT* in pCold II and self-ligation to exclude the two-third 3′-region: one PstI site in the ORF, the other in a multiple cloning site of the vector. All plasmid DNAs were purified on a spin prep column (Qiagen) for transformation of *Escherichia coli* [E. cloni 10G, Lucigen and BL21(DE3) from Novagen or Delphi Genetics] and by a Qiagen-tip for transfection of HEK cells. DNA sequences were confirmed by a Big Dye terminator mix and an ABI 3130 Sequencer (Applied Biosystems) and later by Fasmac Co Ltd. The primers for PCR are listed in Supplementary Table S1.

### Cell culture, transfection and selection of cells

The HEK (human embryonic kidney)-293T cell line (obtained from Dr K. Murata, Iwaki Meisei Univ.) and its derivative T-Rex293 (HEK-TR, Life Technologies), were cultured in a MegaCell Dulbecco's Modified Eagle's Medium (Sigma) with 3% FBS, 2 mM glutamine, 50 μg/ml gentamycin in an atmosphere of 95% air/5% CO_2_ at 37°C. For the generation of HEK-TR cell lines that constitutively express His–BCNT, the cDNA in pCMV6-AN–His was transfected with GenJet (SignaGen Laboratories) and the cells were subjected to limited dilution after 48 h and cultured in the same medium supplemented with 250 μg/ml G418 (Enzo Life Sciences) in a 96-well dish. Among three generated clones, the G11 (HEK-TR clone which constitutively expresses His–BCNT) clone was used for further studies based on the advantages of its growth rate. The clone was routinely sub-cultured in G418-containing medium using Trypsin/EDTA (Sigma).

### Preparation of His–Bcnt from *E. coli*

Various Bcnt in pCold II in *E. coli* were expressed by shifting the temperature to 15°C, letting the cultures stand for 30 min, supplementing with 0.1–1 mM IPTG and then shaking for 19–23 h at 15°C. Target proteins were isolated at 4°C basically according to the manufacturer's instructions (Qiagen). The cells were lysed in lysis buffer [50 mM sodium phosphate (pH 8.0), 300 mM NaCl, 10 mM imidazole and protease inhibitors] supplemented with lysozyme (1 mg/ml, Sigma) at 4°C for 30 min in a rotary shaker and then sonicated in an ice-water bath for 2.5 min (10-s intervals × 15; Bioruptor, Cosmo Bio.). The extract was centrifuged for 30 min at 4°C at 17000 ***g*** and the supernatant was mixed with Ni-NTA (Ni^2+^-nitrilotriacetate) agarose beads or its magnetic beads (Qiagen) in a siliconized tube and incubated for 1 h in a rotary shaker. Following the removal of the unbound fraction by brief centrifugation, the beads were suspended in lysis buffer and transferred to a spin column using a wide-bore tip. After washing two times with the lysis buffer and then two times with washing buffer containing 20 mM imidazole, the bound proteins were eluted with 150 mM imidazole and then with 250 mM imidazole-containing buffer without any inhibitors.

### Purification of His–BCNT from the G11 clone

The G11 clone was seeded at a density of 1.1 × 10^7^ cells per 150 mm dish (Sigma) and cultured for 3 days with one medium change. To detect acetylation sites, the cells were incubated with 4 mM nicotinamide and 50 nM trichostatin A for 2 h prior to extract preparation. The cell layers were washed once with chilled PBS on ice and harvested in 1 ml of lysis buffer per dish [20 mM Hepes/NaOH (pH 7.5), 150 mM NaCl, protease inhibitors, deacetylase inhibitors and phosphatase inhibitors (1 mM sodium vanadate, 10 mM sodium fluoride, 25 mM β-glycerophoshate)] on ice. The extract was harvested using a scraper, frozen immediately in liquid nitrogen and stored at −80°C until use. For the isolation of His–BCNT, the stored extract was thawed, sonicated and centrifuged as described for the preparation from *E. coli*. The supernatant was incubated with anti-His antibody-linked beads (MBL) in a siliconized tube for 1 h in an ice-water bath. The tube was tipped several times during incubation. The bound fraction was transferred to a spin column in lysis buffer containing all inhibitors. The column was washed three times with lysis buffer with all inhibitors, followed by one wash with PBS without inhibitors, after which the adsorbed fraction was eluted with 50 μl of 2 mM His-tag peptide (MBL) at room temperature according to the manufacturer's protocol. Alternatively, the cell extracts were lysed in lysis buffer containing 1% Nondet P-40 (Sigma) and the supernatant was obtained as described above. The extract was fractionated by 20%–45% ammonium sulfate precipitation, after which the pellet was resuspended in lysis buffer with all inhibitors and dialysed overnight against 20 mM Hepes/NaOH (pH7.4), 150 mM NaCl, 1 mM EDTA in a cold chamber. His–BCNT was isolated from the dialysates as described above. Both the N- and the C-terminal regions of BCNT were routinely confirmed by mass analysis using Asp-N (endoproteinase which selectively cleaves peptide bonds N-terminal to aspartic acid and glutamic acid residues) digests to identify *m/z*=MHHHHHHAIAMEEF (*m/z*=1763.6) or MHHHHHHAIAM (*m/z*=1358.5) and DLRLSKMKP (*m/z*=1087.6).

### *In vitro* acetylation assay

Human His–BCNT and two bovine His-variants were isolated from *E. coli* and acetylated using recombinant CREB-binding protein (CBP, 2 μg) and ^14^C-radioactive acetyl-CoA (0.3 μCi, specific activity 3.7-4.44 GBq/μmol, ARC0554) as described previously [15]. The CBP was prepared using a baculovirus-insect cell systems and the details will be described elsewhere (Yasuda, T., et. al., unpublished work). The reaction mixtures contain 50 mM Tris/HCl (pH 8.0), 1 mM EDTA, 1 mM DTT, 10 mM sodium butyrate and 10% glycerol supplemented with deacetylase inhibitors in a total 15 μl. Pol β (2 μg, Bio Academia) and BSA (2 μg) were employed as a positive and negative controls respectively. Following incubation at 30°C for 60 min, the reaction was stopped by the addition of 5× SDS/PAGE sample buffer. The samples were heated at 95°C for 2 min and subjected to SDS/PAGE (4%–20% gradient gel, Bio-Rad) at a constant current of 20 mA. After staining with Coomassie Brilliant Blue (CBB), the gel was soaked in Amplify (GE Healthcare) for 30 min, dried and exposed to X-ray film (Kodak BioMax MR) at −80°C for 1 week.

### Protein phosphatase treatment

Recombinant FLAG-tagged PPM1A (protein phosphatase 1A) and PP2A (protein phosphatase 2A) were isolated from the transfected HEK293 cells essentially as described previously [16]. PP2A was prepared by the co-expression of the haemagglutinin (HA)-tagged PP2A catalytic subunit and the FLAG–PP2A regulatory subunit, followed by isolation with anti-FLAG beads. His–BCNT from the G11 clone was isolated by eluting the affinity beads of the bound fraction with 50 mM glycine buffer (pH 2.7) followed by adjustment to neutral pH with Hepes/NaOH (pH 7.9). The preparation was incubated with PPM1A (30 milliunits) or PP2A (50 milliunits) in the presence or absence of okadaic acid at a final concentration of 100 nM. The reaction mixtures were 50 mM Hepes/NaOH (pH 7.5), 2 mM MnCl_2_ and 0.02% (v/v) 2-mercaptoethanol. After 30 min incubation at 30°C, 5× SDS/PAGE sample buffer was added, after which the samples were boiled immediately and subjected to immunoblot analysis. One unit of phosphatase activity was defined as described [16].

### Immunoblot analysis

Western blotting analysis was carried out essentially as described previously [17] except for two reagents: the second antibody of Clean-Blot (Thermo Fisher Scientific) for guinea pig anti-BCNT-C antibody and the substrate of the nitro blue tetrazolium/5-bromo-4-chloro-3-indolyl phosphate (NBT/BCIP) solution (Roche) or its tablets (Sigma) for development. For reprobing, the filter was treated with 2% SDS, 80 mM 2-mercaptoethanol in 62. 5 mM Tris/HCl (pH7.4) at 50°C for 30 min. After rinsing with H_2_O, the filter was dipped twice in dimethylformamide for 10 s at room temperature, rinsed again with H_2_O and subjected to blocking as usual. An anti-BCNT-C peptide antibody [17] and an anti-6× histidine monoclonal antibody (9C11, Wako) were used.

### NMR study

Production of a ^15^N-enriched BCNT fragment, 40 aa in length (218–257), was carried out using the deca-histidine-tagged ubiquitin fusion system [18]. After isolation by nickel-chelating affinity chromatography, cleavage by yeast ubiquitin hydrolase and HPLC purification with Sep-Pak C18, the sample was dissolved in NMR sample buffer [50 mM sodium phosphate (pH 6.0), 100 mM NaCl, 5 mM DTT, 10% ^2^H_2_O]. The ^1^H-^15^N HSQC spectrum [19] was recorded on a Bruker AMX-500 spectrometer at 15°C. Data processing was performed on a Bruker X-32 UNIX workstation with UXNMR software.

### SDS/PAGE

SDS/PAGE was performed essentially as described by Blattler et al. [20] except that the Tris/glycine buffer system was used [4]. Tricine–SDS/PAGE was carried out as described previously [21]. Two SDS/PAGE molecular mass standard markers, unstained and prestained (Bio-Rad 161-0304 and 161-0373 respectively) were used and the gels were stained with Quick-CBB Plus (Wako).

### Mass spectroscopy analysis

The protein bands were excised and digested with trypsin (TPCK-treated, Worthington Biochemical) or endoproteinase Asp-N (Roche Diagnostics) as described previously [22]. *Achromobacter* protease I (a gift from Dr T. Masaki, Ibaraki University, Ibaraki) [23] was also used in one certain case. The digests were analysed by nano LC–MS/MS using a Q Exactive mass spectrometer (Thermo Fisher Scientific). The database search was performed against an in-house database using the MASCOT program (version 2.3; Matrix Sciences) with variable modifications: Gln → pyro-Glu (N-term Q), Oxidation (M), Phospho (ST), Acetyl (K). Quantitative analysis using Qual Browser (version2.2; Thermo Fisher Scientific) was performed as described previously [22]. The digests were subjected to MALDI–TOF MS analysis using ultrafleXtreme TOF/TOF MS (Bruker Daltonics). The measurement was performed in reflector mode using α-cyano-4-hydroxycinnamic acid as the matrix. The protein C-terminal peptide and probable phosphorylated peptide were analysed by MS/MS in LIFT mode. The localization of certain phosphorylation sites was evaluated using phosphorRS (Proteome Discoverer, v. 1.4.0.288, Thermo Fisher Scientific). The MS proteomics data have been deposited to the ProteomeXchange Consortium [24] via the PRIDE partner repository (http://proteomecentral.proteomexchange.org) with the dataset identifier PXD002228.

## RESULTS

### An acidic stretch in the N-terminal region is mainly responsible for unusual mobility on SDS/PAGE

Computer modelling [25] predicts that human BCNT is largely disordered (Figure 1A). We evaluated this prediction by NMR analysis of the 40 aa terminal region of BCNT-C (Figure 1B), which the program suggests is mostly disordered. The ^1^H-^15^N-HSQC spectrum shows that most of the ^1^H frequencies of the backbone amides are clustered within a ∼1 ppm area with extensive resonance overlap. The result indicates that the fragment is largely unstructured [26] supporting the prediction. Mammalian Bcnt appears at ∼50 kDa on SDS/PAGE despite its calculated molecular mass of ∼33 kDa. To characterize this discrepancy, we expressed various truncated human or bovine Bcnt as N-terminal His-tagged proteins in *E. coli* and examined the relationship between the estimated mass on SDS/PAGE and the calculated mass (Figure 2; Supplementary Figure S1). The data show that the N-terminal region (177 aa in human BCNT, 175 aa in bovine Bcnt, 173 aa in mouse and rat Bcnt; Figure 3A) is mainly responsible for the mobility shift on SDS/PAGE. Several lines of evidence suggest that acidic aa residues in the intrinsically disordered region cause the unusual electrophoretic behaviour [27–29]. Since the disordered N-terminal region of BCNT contains an acidic stretch (14 acidic aa in region of aa 71–92; 64%), including stretches of seven and five consecutive acidic aa residues (Figure 3A), we evaluated the effects of this acidic stretch on SDS/PAGE mobility by generating three deletion mutants lacking one or both acidic aa stretches. All these mutants show significantly increased mobility; the double deletion mutant Δ(A+B) appears at 39 kDa (Figures 3B and 3C), whereas its calculated mass is 33.4 kDa, indicating a 12 Δ% (percent difference between the estimated from SDS/PAGE and calculated masses). We further examined the effect of one or two acidic residues in the more N-terminal acidic stretch by generating swap mutants substituting human (H) EEEEEE into bovine (B) EEDEE, mouse (M) EEEE or rat (R) DEEE (Figure 3A). The swap mutants H-M and H-R run significantly faster than H-B (Figure 3D), indicating that the acidic aa residue(s) in this stretch have an effect on SDS/PAGE mobility. We conclude that the acidic stretch in the N-terminal disordered region of Bcnt is a main factor in the anomalous mobility of the protein in SDS/PAGE.

**Figure 1:**
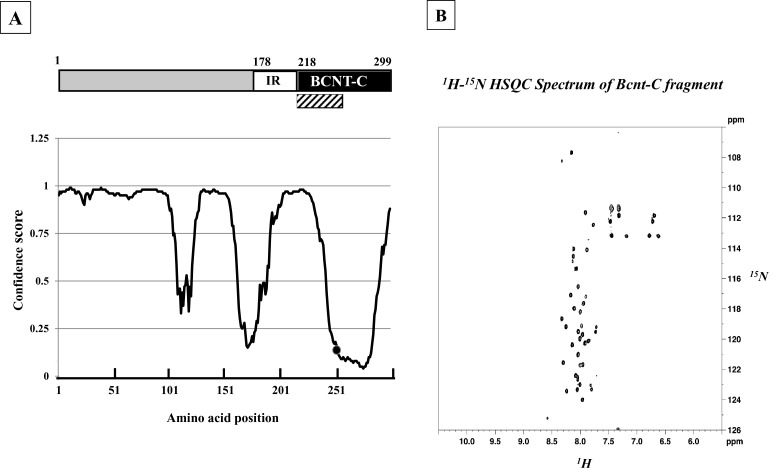
Bcnt/Cfdp1 with largely disordered structure (**A**) Molecular architecture of human BCNT/CFDP1. The protein comprises an acidic N-terminal region, lysine/glutamic acid/proline-rich 40 aa stretch (IR) and a highly conserved C-terminal region (BCNT-C). The aa sequences were analysed using UCL software [25]; confidence scores were converted using Excel (Microsoft, Version 14.5.0) to obtain a specific disordered disposition profile with the horizontal axis indicating aa position. • indicates the position of Ser^250^. A disorder disposition greater than 0.5 in more than 50% of the protein indicates a disorder propensity. The hatched box indicates the 40-aa fragment subjected to NMR analysis. (**B**) ^1^H-^15^N HSQC spectrum. NMR analysis was performed on a ^15^N-labelled 40 aa fragment of BCNT-C (hatched box).

**Figure 2:**
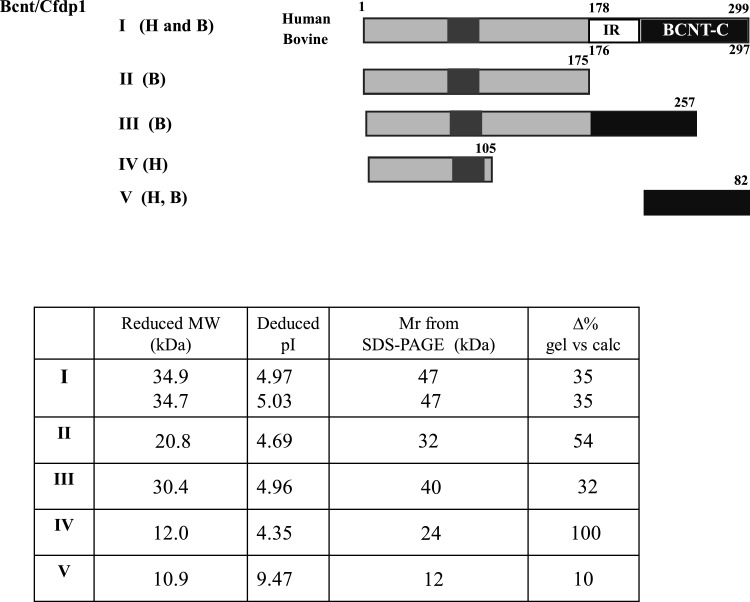
Relationship between apparent and calculated molecular masses of various Bcnt molecules Various human (H) or bovine (B) Bcnt proteins schematically shown at the top were expressed as N-terminal His-tagged proteins in *E. coli.* V (H, B) indicates identical BCNT-C domain between human and bovine. The proteins were isolated using nickel-agarose and analysed by SDS/PAGE (Supplementary Figure S1). The calculated molecular mass and *M*_r_ from the gel including the His-tag peptide MNHKVHHHHHH (1.45 kDa), as well as the isoelectric points (pI) are shown. The column, Δ% gel compared with calc, represents the percent difference between the calculated molecular mass and *M*_r_ estimated by SDS/PAGE.

**Figure 3:**
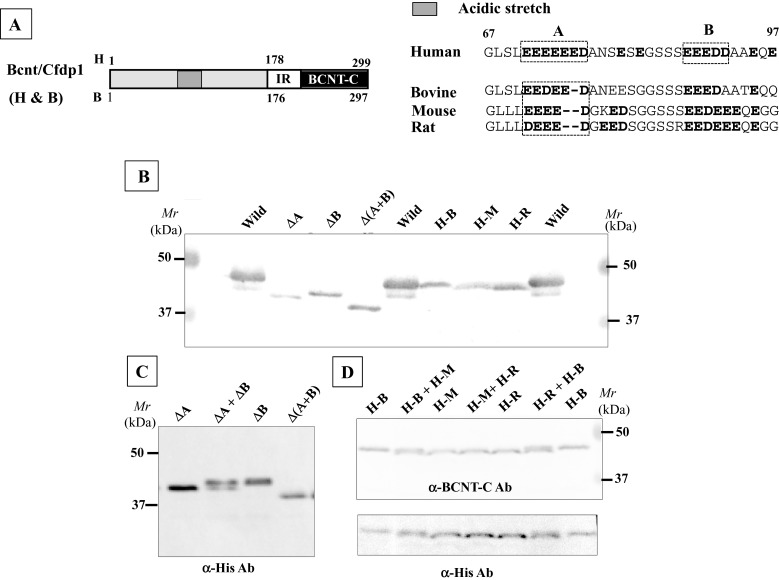
Acidic stretch in the N-terminal region responsible for anomalous mobility on SDS/PAGE (**A**) Acidic stretches in the N-terminal regions of various Bcnt. The aa sequences including the acidic stretch of human (71–92), bovine, mouse and rat are aligned. (**B**) Influence of the acidic stretch on SDS/PAGE mobility. Three BCNT mutants with acidic stretches deleted, ΔA, ΔB and Δ(A+B) and three swapped Bcnts, H-B, H-M and H-R, were expressed as His-tagged proteins in *E. coli*. The former three mutant proteins were isolated, whereas the latter three were cell extract preparations. All mutants were separated on SDS/PAGE and subjected to immunoblot analysis with anti-BCNT-C antibody (Ab). (**C** and **D**) Significant difference in mobility. To show the differences in mobilities, mixed samples as shown at the top of each lane were also separated and detected by immunoblotting with anti-His-tag Ab (**C**) and anti-BCNT Ab, followed by reprobing with anti-His-tag Ab (**D**).

### Exogenous BCNT appears as a doublet when expressed in HEK clone

To examine the effects of PTMs on the mobility shift in SDS/PAGE, we generated HEK-TR cell lines that constitutively express His–BCNT. Although a major band at 47 kDa appears following transient expression, the established clone stably expresses a doublet at 49 and 47 kDa (Figure 4A; Upper and Lower, hereafter). To clarify the difference between the two bands, we isolated the doublet from the G11 extract using anti-His-tag beads (Figure 4B). The yield of the affinity-bound target was approximately one-third in both the presence and the absence of a non-ionic detergent in the cell extract preparation. To improve the yield, the extract was fractionated by ammonium sulfate precipitation followed by dialysis prior to binding to the affinity beads, but this resulted in the isolation of the only Lower band with a similar yield (Figure 4C). Although a few non-specific bands cross-reacted with the affinity beads, the isolate was suitable for further mass analysis.

**Figure 4:**
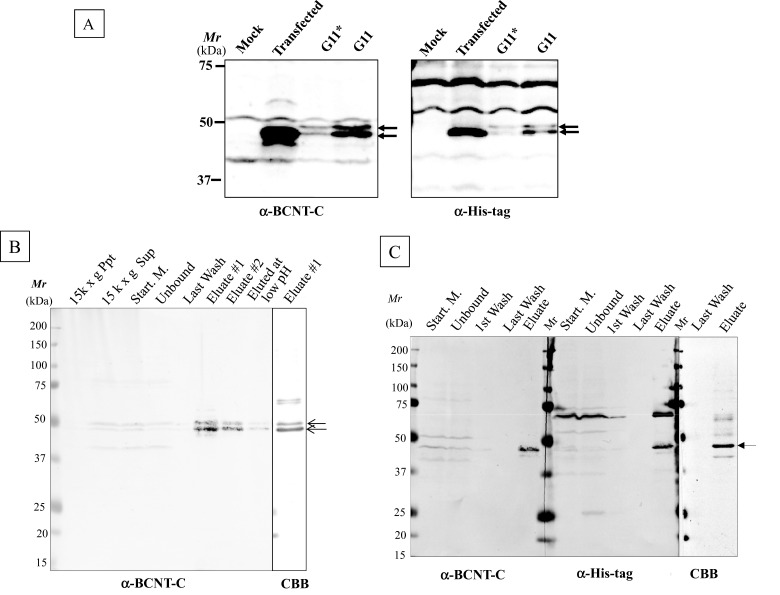
His–BCNT doublet in an established clone and its purification (**A**) Exogenous BCNT expressed in transient and established clones. Human *BCNT* in the pCMV6-AN–His vector was transfected into a derivative of the HEK293T cell line, HEK-TR and the extracts of the transiently expressed cells after 40 h (transfected) or the established clone (G11) were analysed by immunoblotting with anti-BCNT-C Ab (antibody) or anti-His-tag Ab. G* indicates half levels of G11, the extract equivalent to 8 × 10^4^ cells. (**B** and **C**) Isolation of His–BCNT from the G11 clone. His–BCNT was purified from an extract of the G11 clone in two ways. The supernatant of the extract was incubated directly with anti-His-tag antibody beads and the adsorbed fraction was eluted with His-tag peptide (**B**). Alternatively, prior to mixing with the affinity beads, the extract was subjected to ammonium sulfate fractionation and His–BCNT from the dialysates was isolated as in **B** (**C**). Each fraction was separated by SDS/PAGE and detected by immunoblot analysis or CBB staining.

### BCNT is highly phosphorylated including at a unique Ser^250^ in BCNT-C

Phosphorylation sites were examined by MALDI–TOF MS and LC—MS/MS analysis using the in-gel digests with both Asp-N and trypsin (Supplementary Table S2 and PXD002228). A total of seven phosphorylation sites were identified without any pre-treatment (Figure 5B). Mass quantitative analysis revealed three sites, Ser^21^, Ser^26^ and Ser^250^ that are heavily phosphorylated, although Ser^250^ phosphorylation is apparent only in Upper band (Figure 5A, middle column; Supplementary Figure S2). The BCNT from a dialysed preparation shows the same phosphorylation pattern as Lower band including the lack of Ser^250^ phosphorylation (Figure 5A; compare the top and third columns). On the other hand, four acetylated lysine residues were identified only when the extract was prepared from the G11 clone pre-cultured with deacetylase inhibitors as shown below (Figure 9).

**Figure 5:**
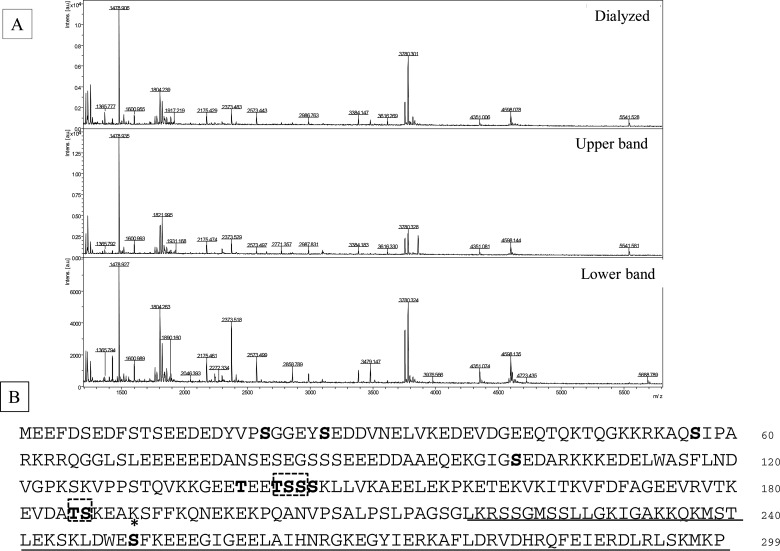
His–BCNT phosphorylated sites *in vivo* including a unique Ser^250^ in Upper band (**A**) Unique Ser^250^ phosphorylation in Upper band. His–BCNT isolated from the G11 clone extract was subjected to MALDI–TOF mass analysis of in-gel digests with Asp-N: Dialysate (top), Upper band (middle) and Lower band (bottom). The peak indicated by the arrow in Upper band is a *m/z*=3860 fragment corresponding to the sequence DWE(p)SFKEEEGIGEELAIHNRGKEGYIERKAFL that includes ionized phosphorylated Ser^250^. (**B**) *In vivo* phosphorylation sites. Phosphorylated sites are shown in bold letters and * indicates the Ser^250^ phosphorylation site specific to Upper band. The dashed boxes indicate that one of the residues is phosphorylated in the region and the underlined region represents the BCNT-C domain. Detailed data are shown in Supplementary Table S2, Supplementary Figures S2 and S3 and PXD002228.

### Phosphatase treatment converges Upper band with Lower band

To evaluate the effect of phosphorylation on SDS/PAGE mobility, we isolated and treated the doublet with recombinant PPM1A or PP2A phosphatase in the presence of Mn^2+^. Incubation with these two phosphatases causes Upper band to converge with Lower one and okadaic acid, a PP2A-specific inhibitor, blocks this shift (Figure 6). The result strongly suggests that Ser^250^ phosphorylation contributes to the mobility difference between the two bands on SDS/PAGE.

**Figure 6:**
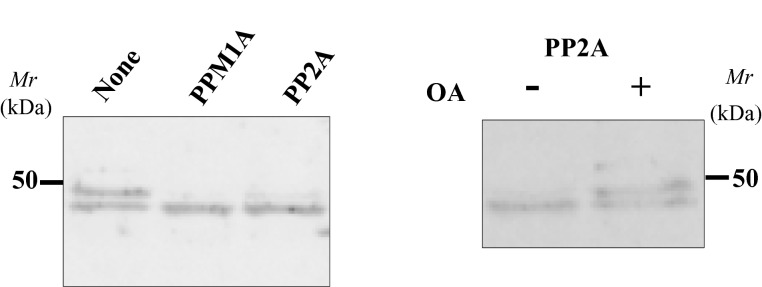
Phosphatase treatment of the isolated His–BCNT doublet Treatment with phosphatases: Isolated His–BCNT from the G11 clone was incubated with the recombinant phosphatases rPPMIA or rPP2A (left panel) or with rPP2A in the presence or absence of okadaic acid (right panel). After 30 min incubation, the samples were boiled in SDS/PAGE sample buffer and subjected to immunoblot analysis with anti-BCNT-C Ab (antibody).

### Ser^250^ phosphorylation significantly contributes the SDS/PAGE mobility shift

Among the seven phosphorylation sites, Ser^250^ resides in a unique Trp-Glu-Ser-Phe (WESF) sequence in BCNT-C. We replaced the serine with alanine or glutamic acid and expressed the two mutants in either *E. coli* or HEK-293T cells. All of the mutant proteins show distinctively different mobilities on SDS/PAGE, indicating that Ser^250^ phosphorylation contributes significantly to the mobility shift (Figure 7).

**Figure 7:**
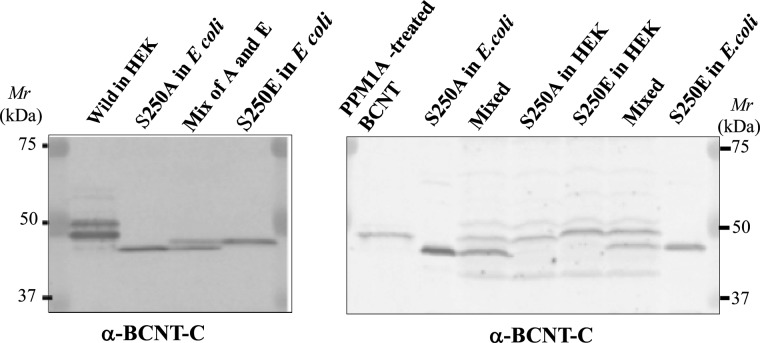
Effects of aa substitutions at Ser^250^ in His–BCNT on SDS/PAGE mobility Influence of substitution of Ser^250^, S250A and S250E mutants of His–BCNT were expressed in *E. coli* or transfected in HEK293T cells for 36 h. Each extract or its mixture as shown at the top of each lane was subjected to SDS/PAGE with detection by immunoblotting with anti-BCNT-C Ab (antibody).

### BCNT is well acetylated by CBP *in vitro* including the *in vivo* acetylation sites

The yeast Swc5 and *Drosophila* Yeti homologues may function as chaperones for the Swi–Snf complex [3,8]. Since Swi–Snf has been identified as a binding protein of a transcription co-activator CBP/p300, as Snf2-related CBP activator protein (SRCAP) [30], the acetylation of BCNT as mentioned above is probably caused by CBP/p300 acetyltransferase. We tested this possibility by *in vitro* acetylation using a recombinant CBP and three Bcnt variants isolated from *E. coli* as substrates (Figure 8A). The radioactivities from ^14^C-acetyl CoA incorporated into all three Bcnt proteins were comparable to that of DNA polymerase β, a known acetylation substrate [15] (Figure 8C), indicating that Bcnt is a good substrate for *in vitro* acetylation by CBP. Next, we identified these acetylation sites and compared them with the *in vivo* acetylation of His–BCNT from the G11 clone pre-cultured with deacetylase inhibitors prior to extract preparation. Among 15 lysine residues acetylated *in vitro*, four, including Lys^268^ in BCNT-C, were identified as acetylated *in vivo* (Figure 9; Supplementary Table S3; Supplementary Figure S4; and PXD002228).

**Figure 8:**
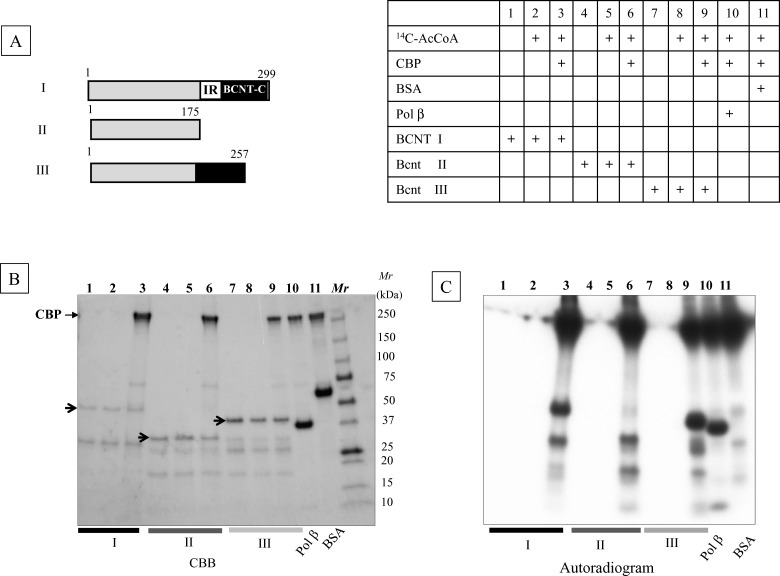
*In vitro* acetylation of Bcnt by CBP (**A**) Components of reaction mixture: Acetylation reaction mixtures containing His-human BCNT (I), -bovine Bcnt (II) or -bovine Bcnt (III), as shown schematically in the left top panel, recombinant CBP and radioactive acetyl-CoA are described in the right top table. Pol β and BSA were used as positive and negative controls respectively. (**B**) Protein pattern of reaction mixtures. Reaction mixtures were separated by SDS/PAGE and the gel was stained with CBB. Three arrows indicate the corresponding non-degraded Bcnt proteins. (**C**) The autoradiogram was obtained by exposing the dried gel shown in (**B**).

**Figure 9:**
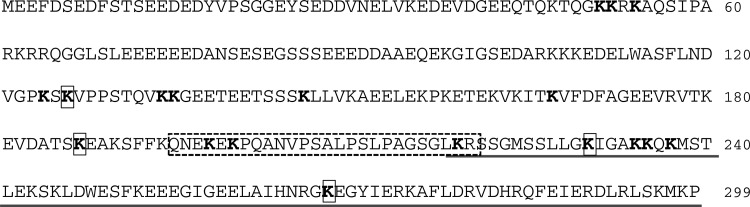
Acetylation sites *in vitro* and *in vivo* *In vitro* acetylation was carried out using His–BCNT and cold acetyl-CoA similar to the experiments shown in Figure 8. His–BCNT was isolated from the G11 clone pretreated with deacetylase inhibitors prior to extract preparation. *In vitro* and *in vivo* acetylation sites were identified by LC–MS/MS analysis of in-gel digests with trypsin or Asp-N as shown in Supplementary Table S3, Supplementary Figures S3 and S4 and PXD002228. Lysine residues acetylated *in vitro* are shown in bold letters; the dashed triangle indicates a region in which one of the three lysine residues is acetylated. The four lysine residues acetylated *in vivo* are shown by open boxes. The underlined region represents the BCNT-C domain.

## DISCUSSION

In the present study, we expressed His–Bcnt proteins carrying various deletions in *E. coli* and HEK cells and found that an acidic stretch in the N-terminal region and Ser^250^ phosphorylation are the main causes of the complex mobility shift on SDS/PAGE. It has been shown that protein mobility on SDS/PAGE is generally dependent on electrostatic effects for cytosolic proteins [31] and hydrophobic and/or conformational effects for membrane protein [32]. In addition, several lines of evidence suggest that adjacent acidic aa residues in disordered regions have distinctive effects on SDS/PAGE mobility [27–29]. Since mammalian Bcnt contains acidic stretches in the highly disordered N-terminal region (Figures 1 and 2A), the protein is ideal to test this premise. We generated three mutants with one or both acidic stretches deleted, as well as three swap mutants within the stretch (Figures 2B–2D) and confirmed the proposition.

To examine the influence of PTMs on SDS/PAGE mobility, we generated a HEK-TR cell line that constitutively expresses His–BCNT that migrates as a doublet of 47 and 49 kDa on SDS/PAGE. We isolated the doublet with a yield of only one-third in either the presence or the absence of a non-ionic detergent during the extract process and in the case of fractionation of the extract or no fractionation prior to affinity purification. Although we tried to recover His–BCNT from the fraction not bound to the resin, no significant recovery was achieved with either anti-His-tag or nickel-affinity beads in the second round. This suggests that a significant fraction of His–BCNT might form complexes with cellular component(s) and/or undergo dimerization that would block access to the N-terminal His-tag for affinity binding, although we are unable to exclude the possibility of a general expression problem concerning the production of intrinsically disordered proteins.

Under these limiting conditions, we identified seven phosphorylation sites without pre-treatment with phosphatase inhibitors, in contrast with the detection of acetylation of exogenous BCNT expressed in HEK-TR cell lines for which pre-culture with histone deacetylase inhibitors is required. Secondary PTMs of exogenous proteins is unavoidable from either transiently or permanently expressing cells. For the transiently expressed BCNT, we were able to detect its acetylation but not phosphorylation. This sample is tagged with a C-terminal Myc and isolated from HEK293 cells pre-cultured for 24 h with sodium butyrate, a histone deacetylase inhibitor, prior to extract preparation and prepared in the presence of sodium vanadate, a phosphatase inhibitor (Dr L. Mull, OriGene Technologies, personal communication). It is interesting to compare the differences in PTMs between transiently and constitutively expressed proteins more precisely taking into account the differences in the epitope tags and their locations at either the N- or the C-terminus.

Among seven phosphorylated sites detected in BCNT, the Ser^250^ phosphorylation is apparent only in Upper band, whereas the phosphorylated state of Lower band, including the missing Ser^250^ phosphorylation, is exactly the same as that of the dialysed protein. The substitution of Ser^250^ with alanine or glutamic acid causes significant changes in the SDS/PAGE mobility. However, S250E BCNT expressed in *E. coli* runs slightly faster than Lower band from HEK-293T cells, suggesting that another phosphorylation(s), such as at Ser^21^ and/or Ser^26^, might also influence mobility. Ser^250^ resides in a region between disordered and ordered regions, rather than in a purely disordered region. This type of phosphorylation site accompanying a SDS/PAGE mobility shift is frequently observed such as in superoxide dismutase 1 (SOD1) [31], p56Lck [33] and p35 [34]. We speculate that a local electrostatic environment(s), such as is present in an acidic stretch, as well as an induced phosphorylated site adjacent to hydrophobic aa in a disordered region or a position between disordered and ordered regions, has the potential to affect the number of bound SDS molecules, thus causing a shift in SDS/PAGE mobility (Figure 10). The residue corresponding to Ser^250^ in the unique WESF motif in mammalian Bcnt is asparagine in the chicken CENP-29 homologue [9] and alanine in the zebra fish RGD motif, leucine rich repeats, tropomodulin domain and proline-rich containing (RLTPR) homologue (GenBank: NP_001093505). Although we are unable, at present, to report the biological relevance of Ser^250^ phosphorylation, it may play a crucial role in mammalian Bcnt-specific signalling modes.

**Figure 10:**
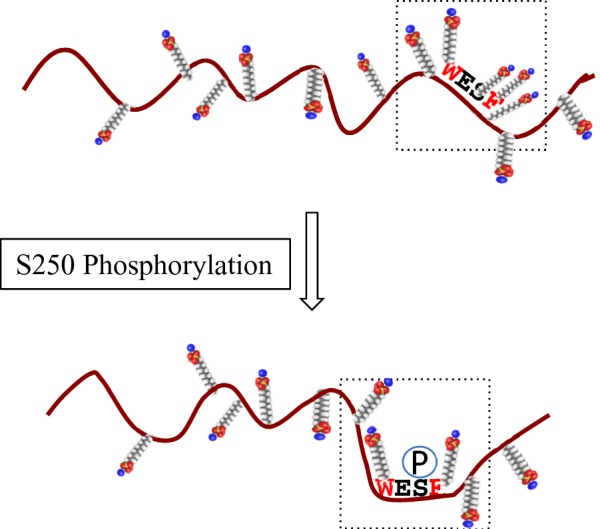
Schematic representation of the effect of Ser^250^ phosphorylation on SDS molecule binding Phosphorylation of Ser^250^, which is located in a disordered region and surrounded by hydrophobic tryptophan and phenylalanine residues, changes the electrostatic environment by reducing the number of SDS molecules bound in the local SDS/protein complex, resulting in a mobility shift on SDS/PAGE. The small molecules represent SDS with the sulfates shown in red.

We previously reported that rat and bovine brain Bcnt appear as doublets at ∼43 kDa [17], which probably corresponds to the His–BCNT doublet described here. The discrepancy in molecular size is caused by the standard molecular markers as shown in Supplementary Figure S1. On the other hand, exogenous human His–BCNT migrates as a more widely separated 53 and 40 kDa (Figure 4) [3], when detected by immunoblot with anti-BCNT-C antibody. So far, we do not know the reason(s) for the generation of doublets from exogenous BCNT expressed in HEK-TR cells that represent two sub-classes with different phosphorylations. In addition, it is still unclear why endogenous BCNT migrates significantly more slowly than Upper band. These are critical subjects to be resolved; it is important to identify other PTMs including ubiquitination [35] and cellular component(s) involved in complex formation related to different phosphorylation site(s). Since human BCNT has been predicted to be a component of a stably-associated soluble complex comprising nine chromatin remodelling-related proteins [10], the present study provides a firm foundation for further studies of its dynamics.

## Abbreviations

aa: amino acid(s)
Asp-N: endoproteinase which selectively cleaves peptide bonds N-terminal to aspartic acid and glutamic acid residues
Bcnt/Cfdp1: Bucentaur/craniofacial developmental protein 1
BCNT-C: the conserved C-terminal region of Bcnt/Cfdp1
CBB: Coomassie Brilliant Blue
CBP: CREB-binding protein
G11: a HEK-TR clone which constitutively expresses His–BCNT
HEK: human embryonic kidney
HEK-TR: a derivative HEK cell of T-Rex-293
IR: intramolecular repeat
PP2A: protein phosphatase 2
PPM1A: protein phosphatase 1A
PTM: post-translational modification
